# A Systematic Analysis Reveals the Prognostic and Immunological Role of Reptin/RUVBL2 in Human Tumors

**DOI:** 10.3389/fgene.2022.911223

**Published:** 2022-06-08

**Authors:** Xiaoru Su, Gaoming Zheng, Zhifang Gui, Xiao Yang, Lahong Zhang, Feng Pan

**Affiliations:** Department of Clinical Laboratory, Affiliated Hospital of Hangzhou Normal University, Hangzhou, China

**Keywords:** reptin, cancer, prognosis, genomic heterogeneity, immune infiltration

## Abstract

Reptin/RUVBL2 is involved in the remodeling of chromatin, DNA damage repair, and regulation of the cell cycle, all of which help to play essential roles in cancer. However, relevant pan-cancer analysis of Reptin is lacking. This study first investigated the potential oncogenic roles of Reptin and revealed a relationship between Reptin with clinicopathological characteristics and immune infiltration based on big data. Here, we showed that Reptin is overexpressed in many cancers. A significant association exists between the expression of Reptin and the prognosis of cancer cases. Reptin had a meaningful interaction with the immune infiltration of CD4^+^ Th1 cells and immune modulator genes in multiple cancer types. And negative correlation exists between Reptin and cancer-associated fibroblasts in BRCA, PRAD, TGCT, and THYM. A significant negative association exists between Reptin and regulatory T cells in TGCT and THCA. Moreover, Reptin is significantly associated with genomic heterogeneity, DNA mismatch repair genes, methyltransferase, and RNA modification genes in specific cancer types. Spliceosome, Hippo signaling pathway, DNA replication pathway, and acetyltransferase activity-associated functions were observed in the effect of Reptin on the tumor. This systematic analysis highlights Reptin as a vital cancer regulator among numerous genes and proved its potential prognosticator value and therapeutic target role for specific tumor types.

## Introduction

Cancer poses the highest social and economic burden of all human diseases, is the second worldwide cause of death, and is a significant barrier to increasing the average lifespan (Mattiuzzi and Lippi, 2019). Despite current therapeutics, including surgery, radiotherapy, chemotherapy, etc., showing certain clinical benefits, the survival rate and prognosis are still deficient. Searching for new tumor molecular markers for cancer diagnosis and therapy is necessary. It is crucial to conduct a comprehensive pan-cancer analysis of a specific gene of interest. The Cancer Genome Atlas (TCGA) database allows us to study the correlation between clinical prognosis and potential molecular mechanisms in pan-cancer.

AAA+ ATPases are a protein superfamily that power various cellular activities. Reptin, also known as RUVBL2, belongs to the AAA+ superfamily and was first reported in the late 1990s in various species, and they share homology with the bacterial RuvB helicase (Mao and Houry, 2017). Research has shown that Reptin is associated with DNA double-strand break repair (Raymond et al., 2015) and participates in the chromatin remodeling complexes INO80 within the nucleus, essential for homologous recombination (Alatwi and Downs, 2015). The ATPase activity of Reptin is required for chromatin remodeling, and the chromatin state further manipulates cell fate (Tarangelo et al., 2015; Blanco et al., 2021). Depletion of Reptin impairs telomerase RNP accumulation (Venteicher et al., 2008). Reptin is also observed to play specific roles in mitosis (Gentili et al., 2015). Reptin is one of the components of the HSP90-interacting chaperone-like complex R2TP (Li et al., 2017b), which can regulate the assembly of small nuclear ribonucleoprotein particles (snRNPs) specific proteins (Malinova et al., 2017). The snRNPs can comprise the spliceosome and mediate the splicing of pre-mRNAs, and RNA undergoing different modifications and splicing could have different biological functions (Matera and Wang, 2014; Gu et al., 2020). As part of the histone acetyltransferase TIP60 complexes, Reptin can also promote E2F1 transcription and regulate the cell cycle (Tarangelo et al., 2015).

In addition, Reptin interacted with bona fide proto-oncogenes, such as c-MYC and β-catenin (Mikesch et al., 2018a; Armenteros-Monterroso et al., 2019). Reptin promotes tumor cell proliferation and progression (Javary et al., 2021; Nakamura et al., 2021). Moreover, *in vivo* silencing of Reptin led to the arrest of tumor progression in human hepatocellular carcinoma xenografts in mice (Menard et al., 2010).

Reptin is a critical and valuable gene. However, studies on the role of Reptin have been confined to specific types of cancer so far. No pan-cancer analysis was found here on the connection between Reptin and multiple cancer types, exploring the common ground based on big data.

The present study comprehensively described the prognostic value and immunological role of Reptin based on the Cancer Genome Atlas (TCGA), Genotype-Tissue Expression (GTEx), cBio Cancer Genomics Portal (cBioPortal), and Tumor Immune Estimation Resource (TIMER). Moreover, the relationship between Reptin and genomic heterogeneity, including Neoantigen (NEO), tumor mutational burden (TMB), microsatellite instability (MSI), DNA mismatch repair (MMR) genes, methyltransferase, RNA modification genes, and related signaling pathways were also evaluated to search for the possible biological mechanism of Reptin in the prognosis or immune within various cancer types.

## Materials and Methods

### Reptin Differential Expression Level Analysis

Reptin expression information from 31 distinct tissues was obtained from the GTEx data (https://commonfund.nih.gov/GTEx). The Reptin level between different cancers and normal tissues was observed by integrating the dataset of TCGA and GTEx in UCSC XENA (https://xenabrowser.net/datapages/), in which clinical follow-up data for cancer patients and RNA sequencing data in TPM format were obtained. All data were processed uniformly by the Toil process (Vivian et al., 2017) and were analyzed after log2 conversion. R software (Version 3.6.3) was utilized to conduct the analysis. The R package “ggplot2 (Version 3.3.3) was used to draw box plots. The Clinical proteomic tumor analysis consortium (CPTAC) dataset from the UALCAN website (http://ualcan.path.uab.edu/) (Chandrashekar et al., 2017) was utilized to conduct Reptin protein-level analysis, in which ten types of tumors: breast cancer (BRCA), ovarian cancer (OV), colon cancer (COAD), clear cell renal cell carcinoma (RCC), uterine corpus endometrial carcinoma (UCEC), lung adenocarcinoma (LUAD), Head and neck squamous carcinoma (HNSC), Pancreatic adenocarcinoma (PAAD), Glioblastoma multiforme (GBM), and Hepatocellular carcinoma (LIHC) were available to conduct the compare between tumor and normal tissue. In addition, Reptin expression levels in pathological stages I-IV of tumors were visualized with violin plots *via* GEPIA.

### Survival Prognostic Analysis

The pan-cancer dataset was downloaded from UCSC website, and the high-quality TCGA prognosis dataset was obtained (Liu et al., 2018). To ensure the reliability of the results, the cancer types for which sample number less than ten were excluded. The links between Reptin and prognosis of cancer cases, including overall survival (OS) and disease-specific survival (DSS) in all TCGA tumors, were investigated by forest plots and Kaplan-Meier survival curves. Univariate survival analysis was conducted to determine the hazard ratios and 95% confidence intervals. The R package maxstat (version: 0.7–25) was used to calculate the best cut-off value for Reptin. The minimum number of grouped samples was greater than 25%, the maximum number was less than 75%, and the patients were divided into high-expression and low-expression groups. All values were transformed with a log2 transformation. The R package (survival, version 3.2–7) was utilized, and a Log-rank test was used for statistical calculation.

### Genetic Variant Analysis

A detailed analysis of genetic alteration frequency, copy number alterations (CNAs), and variations in the types of Reptin genes was investigated on the cBioPortal website (https://www.cbioportal.org/) (Cerami et al., 2012; Gao et al., 2013) across all TCGA tumor types. The mutated site of Reptin is shown in the protein structure sketch map. Additionally, the association between genetic variation of Reptin and the OS of cancer patients was calculated.

### Correlation of Reptin Expression Levels With Neoantigen, Tumor Mutational Burden, Microsatellite Instability, Mismatch Repair, Methyltransferase, and RNA Modification Genes

The standardized pan-cancer data was obtained from the UCSC website. The level 4 simple nucleotide variation dataset of all TCGA tumors was downloaded from the GDC website (https://portal.gdc.cancer.gov/), which was pretreated by MuTect2 software (Beroukhim et al., 2010). R software package maftools (version 2.8.05) was utilized to calculate the TMB of each tumor. MSI scores were obtained from a previous study (Bonneville et al., 2017). All expression values have been transformed by log2. The expression data were applied to study the association between Reptin and mutations in five types of MMRs (mutL homolog 1 (MLH1), mutS homolog 2 (MSH2), mutS homolog 6 (MSH6), PMS1 homolog 2, mismatch repair system component (PMS2), epithelial cell adhesion molecule (EPCAM)). The association between Reptin and DNA methyltransferase 1 (DNMT1), DNA methyltransferase-2 (DNMT2), DNA methyltransferase 3 alpha (DNMT3A), and DNA methyltransferase 3 beta (DNMT3B) were also evaluated. The data of Reptin and three types of RNA modification gene markers, including N1-methyladenosine (m1A), 5-methylcytosine (m5C), N6-methyladenosine (m6A), were extracted from each sample. All normalized values were log2 transformed. Correlation analysis was done by Spearman’s method. Sangerbox tools (http://www.sangerbox.com/tool) were used.

### Immune Infiltration and Immune Checkpoint Gene Analysis

Reptin expression data and the level of immune cell infiltrates were observed in different tumor types *via* TIMER (https://cistrome.shinyapps.io/timer/) (Li et al., 2016; Li et al., 2017a). Two types of immune checkpoint genes [Inhibitory (24 types), Stimulatory (36 types)] were derived from a previous report (Thorsson et al., 2018). All values were log2 transformed. Then Spearman correlation analysis proceeded, and a relevant heat map was created.

### Gene Enrichment Analysis

Based on the STRING database (https://string-db.org/), we downloaded the experimentally determined Reptin-binding proteins. The parameter settings were the following: minimum required interaction score [“High confidence (0.700)”], max number of interactors [“custom value”], and max interactors [“100”] in the first shell. Cytoscape (version 3.7.2) MCODE plug-in was used to visualize the clustered sub-networks, and Cyto-Hubba was applied to determine the top 10 hub genes ranked by Maximal Clique Centrality. Subsequently, GEPIA displayed the top 100 Reptin-interacting genes and performed a Pearson correlation analysis between Reptin and the selected top 10 genes. We converted the expression values to log2 (TPM) for all dot plots. TIMER was utilized to observe the related heat map in the pan-cancer. The overlap between Reptin-binding genes and Reptin-interacting genes is displayed in the Venn diagram. Then the two groups of data, Reptin-binding and Reptin-interacting genes, were utilized to conduct the Kyoto Encyclopedia of Genes and Genomes (KEGG) pathway analysis and Gene Ontology (GO) analysis. Which GO analysis included three parts: Biological Process (BP), Cellular Component (CC), and Molecular Function (MF). These results are shown in the bubble diagram. The “ClusterProfiler” (3.14.3) R (Yu et al., 2012) package was employed for KEGG pathway analysis, and GO annotations were obtained from the “org.Hs.eg.db” (3.10.0) Bioconductor packages for humans. The results were displayed using the R packages “ggplot2 (3.3.3)”.

### Statistical Analysis

The Kruskal-Wallis test was applied to calculate the differential expression of Reptin in normal human tissues. The expression level of Reptin in TCGA tumors and normal human tissues were calculated using Wilcoxon rank-sum test, and the HR and *p*-value of survival analysis were calculated with Univariate Cox regression methods. According to the Reptin expression level, Kaplan Meier analysis was utilized to explore the survival time of tumor patients. Correlation analysis was performed by Spearman’s method or Pearson’s method, and *p* < 0.05 was considered significant.

## Results

### Reptin Differential Expression Analysis

**FIGURE 1:**
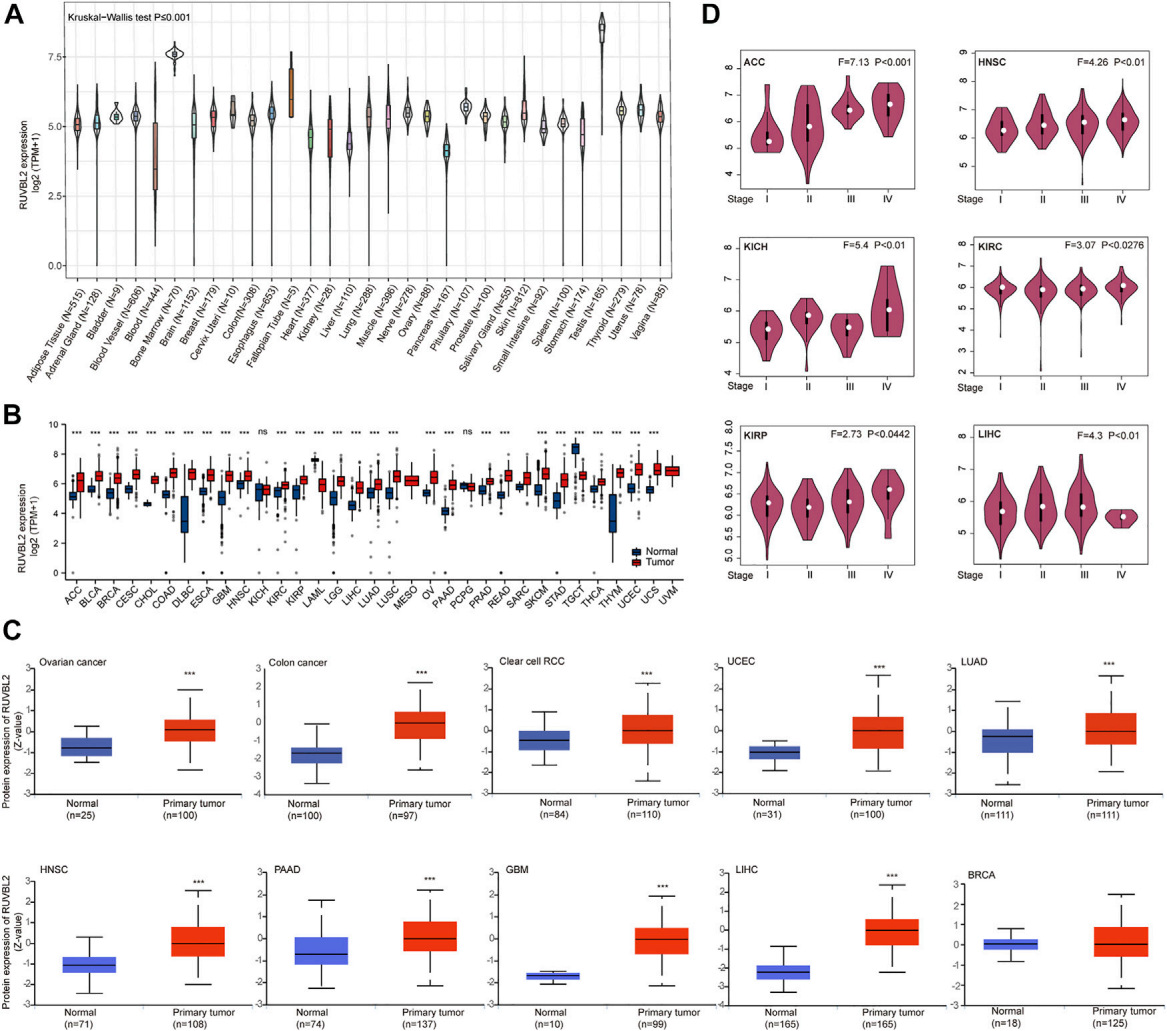
Differential expression of Reptin. **(A)** The expression of Reptin in normal human tissues. **(B)** The expression of Reptin in 33 types of TCGA cancer. **(C)** The protein level of Reptin in BRCA, OV, COAD, RCC, UCEC, LUAD, HNSC, PAAD, GBM, and LIHC. **(D)** The expression of Reptin in pathological stages I-IV of ACC, HNSC, KICH, KIRC, KIRP, and LIHC. *p < 0.05, **p < 0.01, ***p < 0.001.

The mRNA expression, protein level, and different pathological stage expressions of Reptin were analyzed. The results from GTEx showed that differences exist in Reptin expression in various human tissues. Reptin was expressed highest in the Testis tissues and lowest in the Blood (Figure 1A). Subsequently, TCGA and GTEx data were combined to study the expression of Reptin across all TCGA tumor tissues and normal human tissues. Reptin mRNA expression, illustrated in Figure 1B, was significantly higher in tumor tissues compared to normal tissues in the majority of cancers, including adrenocortical carcinoma (ACC), bladder urothelial carcinoma (BLCA), BRCA, cervical squamous cell carcinoma (CESC), cholangiocarcinoma (CHOL), COAD, lymphoid neoplasm diffuse large B cell lymphoma (DLBC), esophageal carcinoma (ESCA), GBM, HNSC, kidney renal clear cell carcinoma (KIRC), kidney renal papillary cell carcinoma (KIRP), brain lower grade glioma (LGG), LIHC, LUAD, lung squamous cell carcinoma (LUSC), OV, PAAD, prostate adenocarcinoma (PRAD), rectum adenocarcinoma (READ), skin cutaneous melanoma (SKCM), stomach adenocarcinoma (STAD), thyroid carcinoma (THCA), thymoma (THYM), UCEC and uterine carcinosarcoma (UCS) datasets. However, the mRNA expression of Reptin was significantly lower in acute myeloid leukemia (LAML) and testicular germ cell tumor (TGCT) datasets. At the same time, there was no difference in kidney chromophobe (KICH), and pheochromocytoma and paraganglioma (PCPG).

The comparison of Reptin total protein levels according to the CPTAC database indicated higher Reptin levels in the tumor tissues of OV, COAD, RCC, UCEC, LUAD, HNSC, PAAD, GBM, and LIHC than those in normal human tissues; however, there was no statistical difference in the BRCA (Figure 1C). GEPIA was applied to study the association between Reptin and different pathological stages of TCGA tumors. The results showed that Reptin expression in stages I–IV of cancer cases, including ACC, HNSC, KICH, KIRC, KIRP, and LIHC, all had *p* values of less than 0.05 (Figure 1D).

### A Prognosis Analysis of Reptin in Pan-Cancer

**FIGURE 2:**
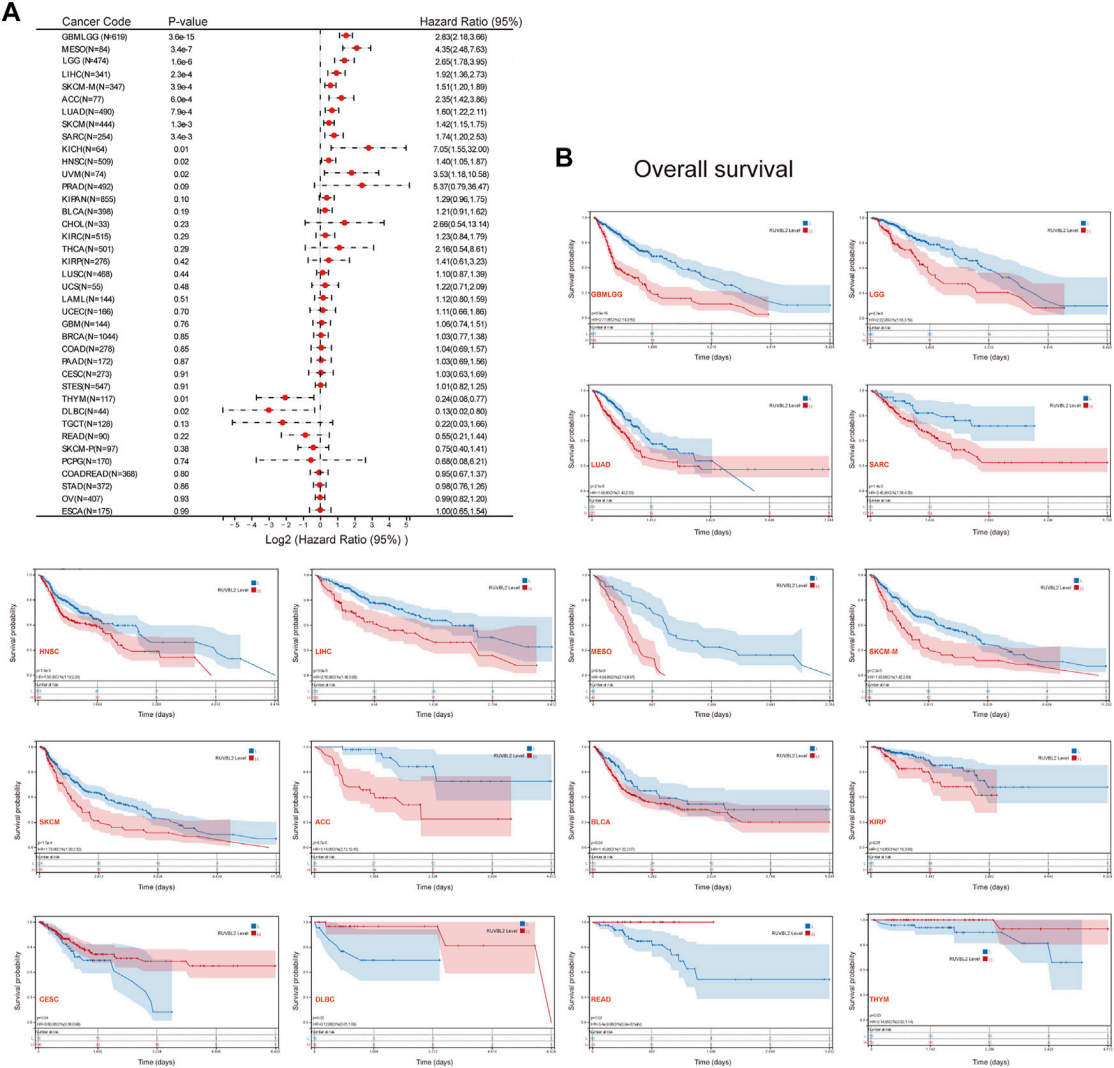
Prognosis analysis of Reptin in pan-cancer. **(A)** Forest plot of the associations between Reptin and OS in 39 types of tumors. **(B)** Kaplan-Meier survival analysis of the relationship between Reptin and OS. Only positive results were presented.

**FIGURE 3:**
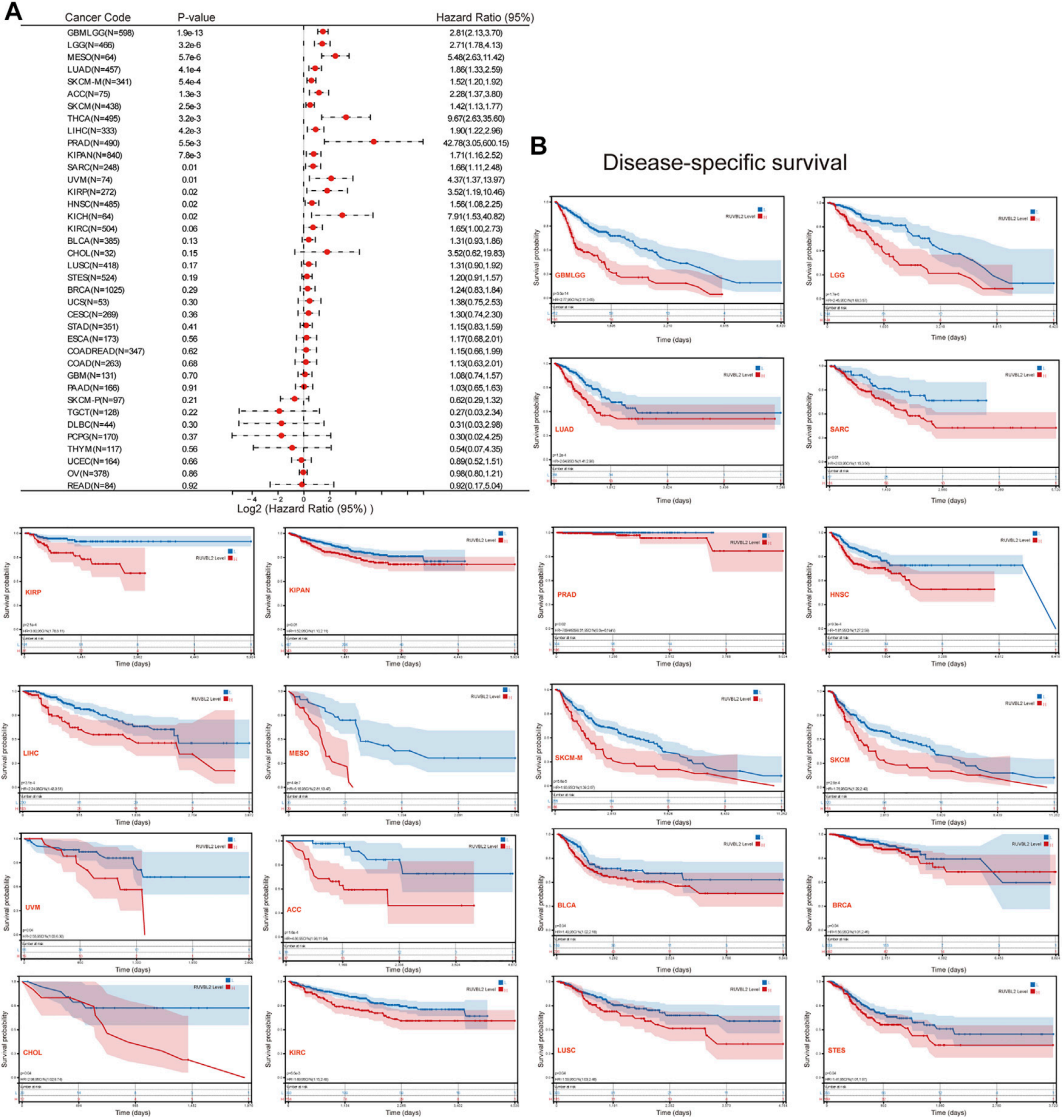
Prognosis analysis of Reptin in pan-cancer. **(A)** Forest plot of the associations between Reptin and DSS in 38 types of tumors. **(B)** Kaplan-Meier survival analysis of the relationship between Reptin and DSS. The positive results are shown.

We used the survival metrics of OS and DSS to estimate the link between Reptin and the prognosis of multiple cancer cases. Cox regression analysis suggested that Reptin is significantly related to OS in Glioma (GBMLGG), sarcoma (SARC), mesothelioma (MESO), uveal melanoma (UVM), LGG, LUAD, HNSC, LIHC, metastatic SKCM (SKCM-M), SKCM, ACC, KICH, THYM, and DLBC (Figure 2A). Besides, Kaplan-Meier survival analyses were conducted based on the expression of Reptin. The results indicated that the upregulation of Reptin was strongly related to worse OS in GBMLGG, LGG, LUAD, SARC, HNSC, LIHC, MESO, SKCM-M, SKCM, ACC, BLCA, and KIRP. In contrast, low expression of Reptin was remarkably related to worse OS in CESC, DLBC, READ, and THYM (Figure 2B). In addition, we explored the link between Reptin and DSS in these types of tumors. Cox regression analysis suggested that Reptin is significantly related to DSS in 16 types of cancer, including GBMLGG, LGG, LUAD, SARC, KIRP, KIPAN, PRAD, HNSC, LIHC, THCA, MESO, SKCM-M, SKCM, UVM, ACC, and KICH (Figure 3A). The Kaplan–Meier survival analyses demonstrated that the upregulation of Reptin was very strongly related to worse DSS in 20 types of cancer, including GBMLGG, LGG, LUAD, SARC, KIRP, KIPAN, PRAD, HNSC, LIHC, MESO, SKCM-M, SKCM, UVM, ACC, BLCA, BRCA, CHOL, KIRC, LUSC, and Stomach and Esophageal carcinoma (STES) (Figure 3B).

### Reptin Genetic Variant Pattern Analysis

**FIGURE 4:**
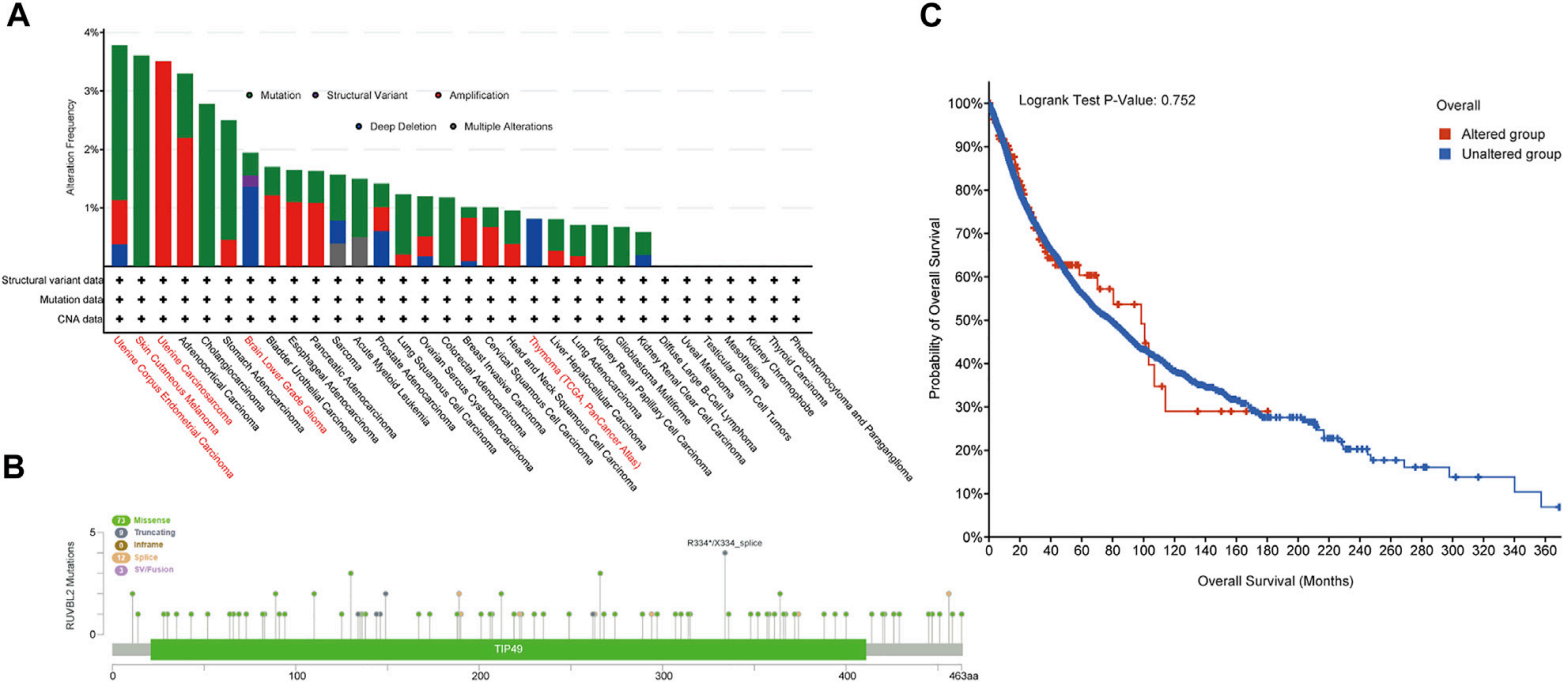
Mutation characteristics of Reptin. **(A)** The frequencies of different genetic variant types in all TCGA tumor samples. **(B)** The distribution of Reptin mutation sites. **(C)** The comparison of clinical survival prognosis analysis between the Reptin altered group and the unaltered group.

The cBioPortal database was applied to observe the genetic variant status of Reptin in TCGA tumors. The results are displayed in Figure 4A. As shown, UCEC patients had the highest frequency of Reptin alteration (∼3.8%), in which mutation was the primary type. The second group was SKCM patients with an alteration frequency of Reptin of about 3.6%, in which every observed case had a mutation. It is worth emphasizing that all UCS cases with genetic variants had amplification of copy number alteration (CNA) (∼3.5% frequency). The copy number deletion of Reptin was the primary type in LGG patients (∼2% frequency). Thymoma cases showed an alteration frequency of less than 1%. Figure 4B displays the case number, types, and sites of the Reptin genetic variants. In short, missense mutation of Reptin was the primary type. Additionally, we explored the possible association between alterations of Reptin and the prognosis of various cancer patients. The results demonstrated that Reptin had no difference in OS between the patients with genetic alterations and patients without alterations (Figure 4C).

### Correlation of Reptin Expression Levels With Neoantigen, Tumor Mutational Burden, Microsatellite Instability, Mismatch Repair, Methyltransferase, and RNA Modification Genes

**FIGURE 5:**
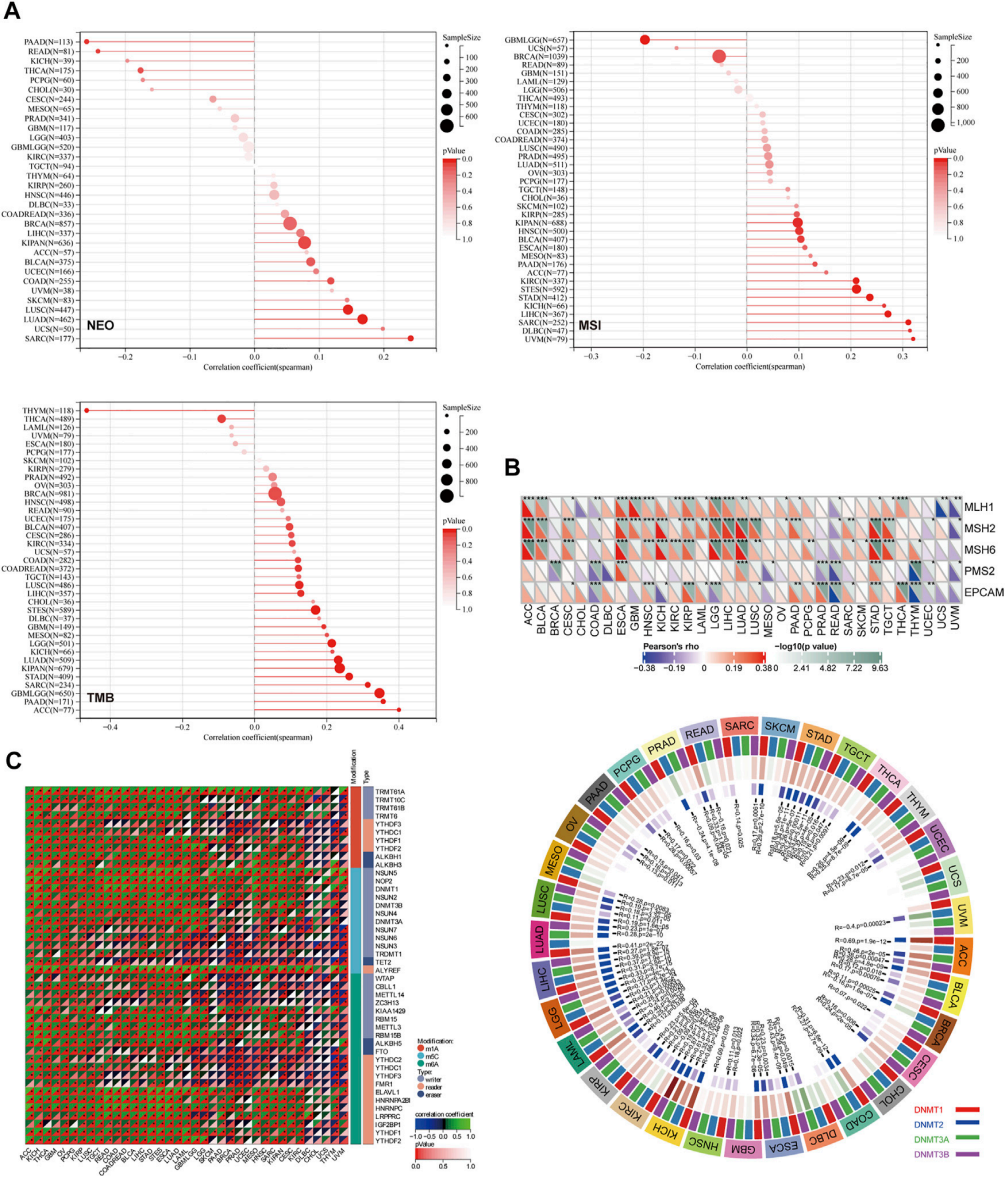
The correlation analysis between Reptin and NEO, TMB, MSI, MMRs, methyltransferase, and RNA modification genes. **(A)** The correlation between Reptin and NEO, TMB, MSI in pan-cancer. **(B)** The correlation between Reptin and MMRs, methyltransferase. **(C)** The correlation between Reptin and RNA modification genes. *p < 0.05, **p < 0.01, ***p < 0.001.

Sangerbox tools were applied to calculate the number of new antigens and determine the association between Reptin and neoantigens. As shown in Figure 5A, the results demonstrated that Reptin is positively related to NEO in LUAD (R = 0.17, *p* = 0.0004), SARC (R = 0.24, *p* = 0.008), LUSC (R = 0.14, *p* = 0.003), and negatively related to NEO in READ (R = −0.24, *p* = 0.04) and PAAD (R = −0.26, *p* = 0.02). A highly significant positive association exists between Reptin and TMB in 15 types of tumors, including GBM (R = 0.19, *p* = 0.02), GBMLGG (R = 0.35, *p* = 8.4e^−20^), LGG (R = 0.21, *p* ≤ 0.001), LUAD (R = 0.23, *p* = 1.1e^−7^), COAD (R = 0.12, *p* = 0.04), COADREAD (R = 0.12, *p* = 0.02), STES (R = 0.17, *p* ≤ 0.001), SARC (R = 0.31, *p* = 9.07e^−7^), KIPAN (R = 0.24, *p* = 4.12e^−10^), STAD (R = 0.26, *p* = 6.73e^−8^), LUSC (R = 0.12, *p* = 0.005), LIHC (R = 0.13, *p* = 0.01), PAAD (R = 0.36, *p* ≤ 0.001), BLCA (R = 0.097, *p* = 0.049), and ACC (R = 0.40, *p* ≤ 0.001), however, significant negative correlations exist between Reptin and TMB in THYM (R = −0.46, *p* = 1.15e-7) and THCA (R = −0.09, *p* = 0.045). The relationship between Reptin and MSI was investigated in TCGA cancer. The results indicated that there were significant positive correlations between Reptin and MSI in 11 types of cancers, including STES (R = 0.21, *p* = 2.06e-7), SARC (R = 0.31, *p* = 4.53e^−7^), KIPAN (R = 0.098, *p* = 0.009), STAD (R = 0.24, *p* ≤ 0.001), HNSC (R = 0.10, *p* = 0.02), KIRC (R = 0.21, *p* ≤ 0.001), LIHC (R = 0.27, *p* = 1.19e^−7^), UVM (R = 0.32, *p* = 0.003), BLCA (R = 0.10, *p* = 0.03), KICH (R = 0.26, *p* = 0.03) and DLBC (R = 0.31, *p* = 0.03), while there was negative correlation between Reptin and MSI in GBMLGG (R = −0.19, *p* = 4.07e^−7^).

Moreover, the relationship between Reptin and five types of MMR mutation (MLH1, MSH2, MSH6, PMS2, and EPCAM) was evaluated. The results are shown in Figure 5B. Reptin is significantly correlated with different MMR mutations in 31 types of cancer, except in CHOL and DLBC. Moreover, the association between Reptin and methyltransferases (DNMT1, DNMT2, DNMT3A, and DNMT3B) was visualized in Figure 5B. We observed that Reptin is strongly related to methyltransferases in 32 types of TCGA cancer, except in UCS.

It is well known that RNA modification is critical for post-transcriptional gene regulation that can be classified as “writers” (installation), “readers” (recognition), and “erasers” (removal). Thus, the Spearman correlations between Reptin and these regulatory genes were calculated. As shown in Figure 5C, we found numerous meaningful associations. Significant positive correlations exist between Reptin and m1A, m5C, and m6A in multiple cancer types. The molecules that interacted the most with Reptin included TRMT61A, ALYREF, and ELAVL1. TRMT61A is positively associated with Reptin as an m1A writer in 35 types of cancer, and ALYREF, as the m5C reader, was positively associated with Reptin in 35 cancer types. ELAVL1 as an m6A reader positively correlates with Reptin in 32 types of cancer. Notably, in CHOL, Reptin was only significantly negatively correlated with m1A writer TRMT10C and m5C writer NSUN3.

### Immune Infiltration and Immune Modulator Gene Analysis

**FIGURE 6:**
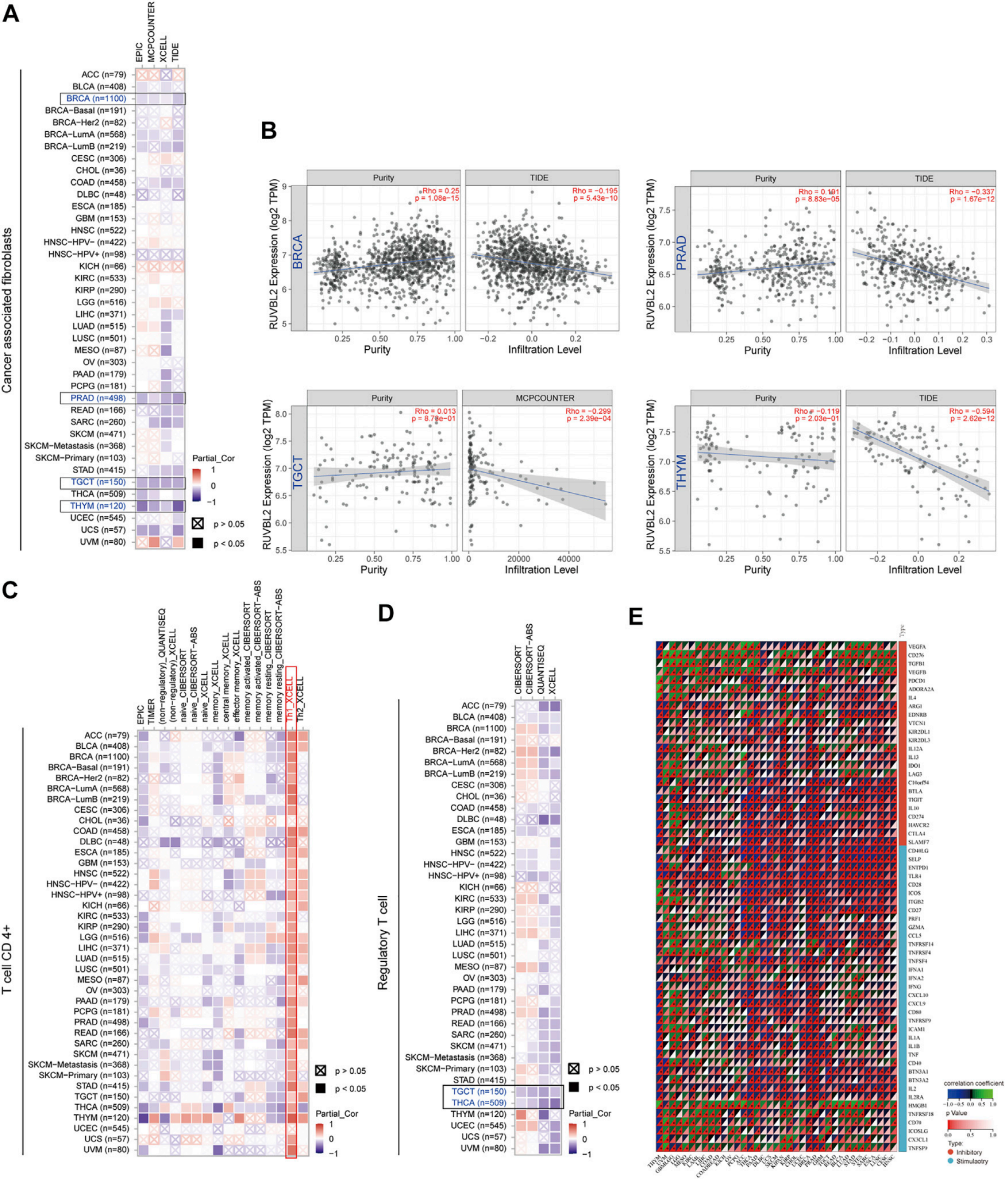
Association between Reptin and immune cell infiltration across all TCGA cancers. **(A)** The association between Reptin and the infiltration of cancer-associated fibroblasts. **(B)** The relationship between Reptin and the infiltration level of cancer-associated fibroblasts in BRCA, PRAD, TGCT, and THYM. **(C)** The correlation between Reptin and the infiltration of CD4^+^ T cell. **(D)** The correlation between Reptin and the infiltration of regulatory T cell. **(E)** The correlation between Reptin and immune modulator genes in pan-cancer. *p < 0.05, **p < 0.01, ***p < 0.001.

We observed a statistically significant negative association between Reptin and the infiltration of cancer-associated fibroblasts in BRCA, PRAD, TGCT, and THYM based on all algorithms (Figure 6A and Figure 6B). Moreover, significant positive correlations between Reptin and infiltration of CD4^+^ Th1 cells were found in most types of tumors, except in CHOL and UCEC, based on the XCELL algorithm (Figure 6C). Besides, there was a statistically negative association between Reptin and regulatory T cells (Tregs) in TGCT and THCA based on all algorithms (Figure 6D).

Furthermore, we extracted sixty immune checkpoint genes and evaluated their correlations with Reptin levels. The results indicated that Reptin levels were significantly negatively related to many immune regulators in most tumors, such as THYM, THCA, PAAD, DLBC, UCS, KIPAN, BRCA PRAD, and GBM. Notably, Reptin levels did not correlate with the sixty immune checkpoint genes in CHOL (Figure 6E).

### Functional Enrichment Analysis of Reptin-Related Partners

**FIGURE 7:**
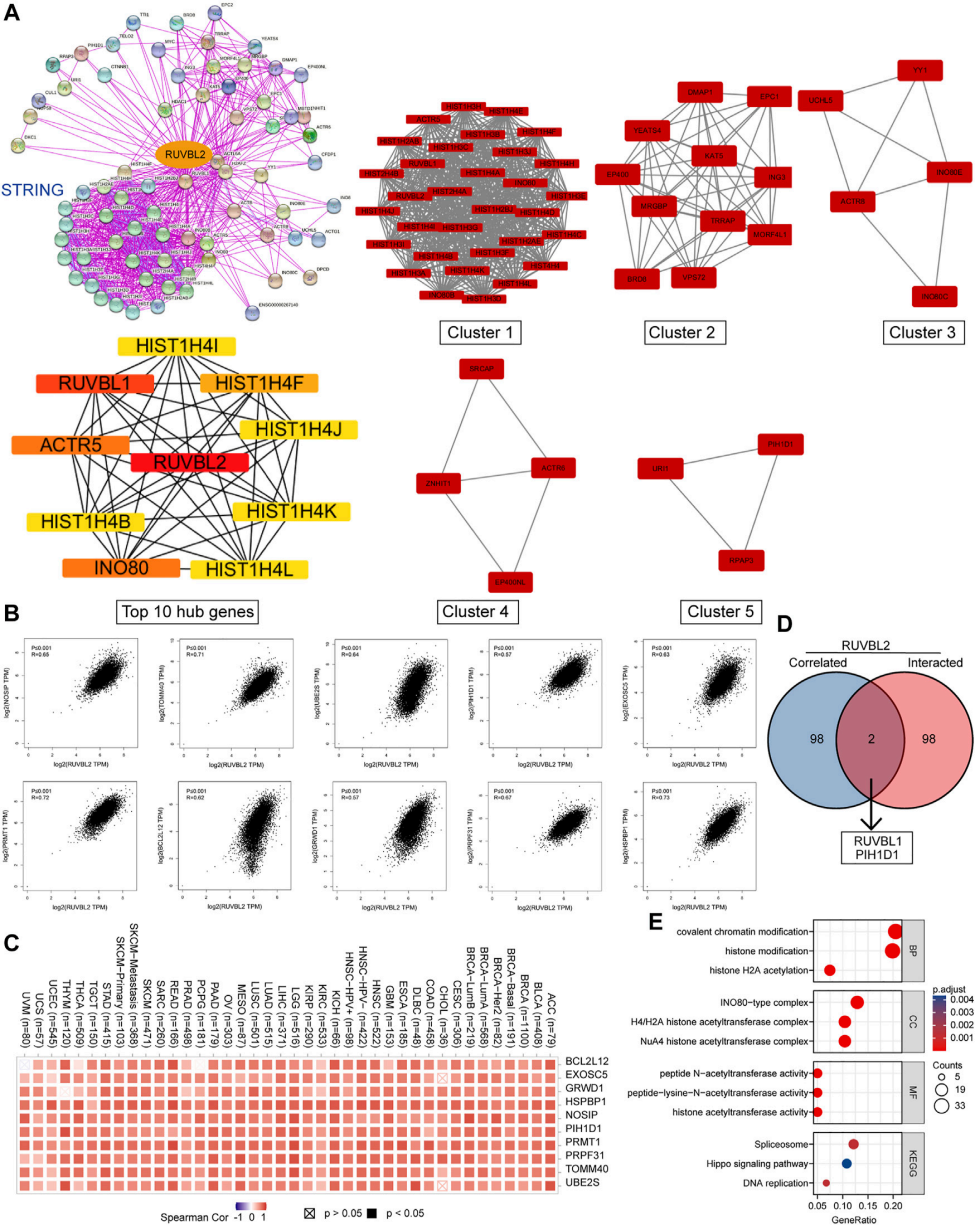
The results of gene enrichment analysis. **(A)** 100 Reptin-binding proteins were shown *via* the STRING tool. Five sub-network modules (Clusters 1–5) and the top 10 hub genes were presented using the Cytoscape plug-in, MCODE, and cytoHubba. **(B)** The association between Reptin and the selected top 10 Reptin-interacting genes. **(C)** The relevant heatmap data in cancer. **(D)** The overlap analysis of Reptin-binding and interacting genes. **(E)** The results of KEGG and GO analysis were visualized.

We screened out the Reptin-binding proteins and Reptin-interacting genes to conduct the functional enrichment analysis. Based on the STRING database, 100 experimental supported Reptin-binding proteins were obtained, and the interaction network was shown in Figure 7A. Highly interconnected five sub-clusters from Cytoscape plug-in MCODE and the top 10 hub genes ranked by Maximal Clique Centrality from Cyto-Hubba were also shown in Figure 7A. Besides, GEPIA was utilized to download the top 100 genes that highly interacted with Reptin. Figure 7B showed that Reptin was significantly positively correlated with HSPBP1 (HSPA (Hsp70) binding protein 1) (R = 0.73), PRMT1 (protein arginine methyltransferase 1) (R = 0.72), PRPF31 (pre-mRNA processing factor 31) (R = 0.67), BCL2L12 (BCL2 like 12) (R = 0.62), GRWD1 (glutamate-rich WD repeat-containing 1) (R = 0.57), NOSIP (nitric oxide synthase interacting protein) (R = 0.65), TOMM40 (translocase of outer mitochondrial membrane 40) (R = 0.71), UBE2S (ubiquitin-conjugating enzyme E2 S) (R = 0.64), PIH1D1 (PIH1 domain containing 1) (R = 0.57), and EXOSC5 (exosome component 5) (R = 0.63), all *p* value less than 0.001. The heat map depicted significant positive correlations between Reptin and the ten genes mentioned above in most tumors (Figure 7C).

The overlap analysis exhibited two common members, RUVBL1 and PIH1D1 (Figure 7D), indicating that RUVBL1 and PIH1D1 are genes that both bind and interact with Reptin. KEGG analysis showed that the spliceosome, Hippo signaling, and DNA replication pathways were involved in the effect of Reptin on the tumor, and the GO study suggested that the majority of these genes were related to acetyltransferase activity (Figure 7E).

## Discussion

The ATPase activity of Reptin is essential for most of its roles in cancer. The role of Reptin in cancer cell migration and invasion has been investigated (Breig et al., 2017). Reptin interacts with p53 and suppresses its anti-tumor activity (Maslon et al., 2010). Targeting Pontin/Reptin can potentially treat cancer (Menard et al., 2010; Mikesch et al., 2018b; Assimon et al., 2019). However, whether Reptin can play a role in the prognosis or immune of various cancers *via* the same mechanisms remains clarified.

To clarify the expression landscape of Reptin, we integrated multiple data across all TCGA tumor types. The study found that Reptin was overexpressed in numerous cancers but not every cancer. It appears downregulated in AML and TGCT. Upregulation of Reptin was significantly related to worse clinical prognosis in most tumors. However, overexpressed Reptin suggested a good prognosis in CESC, DLBC, READ, and THYM.

*In vitro* experiments have already demonstrated the pro-proliferative role of Pontin in oral squamous cell carcinoma (OSCC) (Nakamura et al., 2021), and Pontin overexpression has been described as a poor prognostic indicator for HNSC (Lin et al., 2020). However, relevant analysis about Reptin in HNSC is lacking. Based on the big data, we found that overexpression of Reptin was related to worse prognostic in HNSC. For lung cancer, studies have reported that Excavatolide B may exhibit cytotoxic effects against lung cancer A549 cells by affecting Reptin expression (Velmurugan et al., 2017). Reptin is highly expressed in NSCLC patient tumors, related to poor survival (Mikesch et al., 2018b). Reptin was overexpressed in gastric tissue (Li et al., 2010), and its high expression was related to advanced nodal disease in colon cancer patients (Flavin et al., 2011). For hepatocellular liver carcinoma, overexpressed Reptin is necessary for hepatocyte proliferation (Javary et al., 2021), and it could be used to predict poor prognosis (Yan et al., 2019). Within the excretory system, a previous study showed that overexpressed Reptin is related to the poor differentiation of RCC cells and portends poor outcomes for patients (Ren et al., 2013). These findings outlined above were consistent with our results based on big data. In addition, no reports were found of Reptin on the prognosis of CESC, DLBC, READ, and THYM. Our big data-based study complements this aspect.

Here, we describe a decrease in Reptin expression in AML. Previous results found that Reptin is required for the oncogenic potential of leukemia (Osaki et al., 2013), and silencing Reptin resulted in AML cell apoptosis in engrafted mice by increased transcriptional activation c-MYB (Armenteros-Monterroso et al., 2019). Admittedly, Reptin plays an important role in AML. The level of Reptin in leukemia cells needs to be determined. Although the study found that Reptin is readily detected in MLL-AF9 mouse myeloid cells, they did not compare Reptin levels in leukemia cells and normal blood cells (Osaki et al., 2013).

It should be noted that AML is a “liquid” cancer; the choice of the control, normal tissue, to be compared with the leukemic cells is critical. Our study derived leukemia cells compared with normal blood cells from the GTEx database. Further experiment verification of this point may be required in the future.

In addition, Reptin expression correlated positively with the pathological stage in ACC, HNSC, KICH, KIRC, KIRP, and LIHC. Reptin levels at all four stages seem identical in KIRC and KIRP, with Reptin being most expressed in stage IV cases of KICH. However, there is even a decreased expression of Reptin in stage IV in LIHC, which may be worthy of further study.

We also analyzed the genetic variant pattern of Reptin. The missense mutation of Reptin was the primary type. UCEC patients had the highest frequency of Reptin alteration (∼3.8%). All UCS cases with genetic variants had amplification of copy number, and the copy number deletion of Reptin was the primary type in LGG. Genetic alterations in Reptin did not impact the survival of tumor patients.

Moreover, we provide evidence for the potential functional link between Reptin and NEO, TMB, MSI, MMR, DNA methyltransferase, and RNA modification genes across all TCGA tumors.

Tumor neoantigen, encoded by the tumor cell mutant gene, is highly immunogenic. We can design a new vaccine to achieve the therapeutic effect based on the immune activity of the new antigen (Xu et al., 2020). Our results suggested significant positive correlations between Reptin and tumor neoantigen in LUAD, SARC, and LUSC, and significant negative correlations were found in READ and PAAD. TMB and MSI are related to the immunotherapy response. TMB can be employed in tumor cells to show the number of mutations, and it has been identified as a potential biomarker in advancing targeted therapy (Choucair et al., 2020). Moreover, it can predict the response to immune checkpoint protein PD- L1 treatment (Allgauer et al., 2018). At the same time, the expression of PD-L1 was reported to promote tumor progression and formation (Burks et al., 2019) and enhance the immune evasion potential of cancer (Chu et al., 2022). Studies have shown that high MSI can lead to many molecular changes, including increased expression of neoantigens, high TMB, and abundant tumor-infiltrating lymphocytes (Chang et al., 2018). In the present study, there were negative correlations between Reptin and TMB in THYM and THCA. Positive correlations were observed between Reptin and TMB in GBM, GBMLGG, LGG, LUAD, COAD, COADREAD, STES, SARC, KIPAN, STAD, LUSC, LIHC, PAAD, BLCA, and ACC. Besides, the high expressed Reptin was negatively associated with MSI in GBMLGG and positively related to MSI in 11 types of tumors. These findings indicate that Reptin expression can affect the NEO, TMB, and MSI in specific tumors, thereby changing the patient’s response to immunotherapy.

It is well known that cancer is driven by various genetic and epigenetic alterations, and DNA methylation as a key regulator has a crucial role in epigenetic gene regulation (Gu et al., 2015). DNA methylation can control gene expression without changing the DNA sequence and is involved in phenotypic development and altered susceptibility to disease (Moore et al., 2013; Bauer et al., 2016). We found that Reptin expression was significantly related to methyltransferases expression in 32 tumor types. Reptin was positively correlated with m1A, m5C, and m6A in most tumors. TRMT61A, ALYREF, and ELAVL1 are the most interacting molecules with Reptin. Moreover, loss of the critical gene of the mismatch repair mechanism will cause higher somatic mutations and accelerate tumorigenesis (Maki-Nevala et al., 2019). We found that Reptin expression was significantly related to MMR mutation in 31 tumor types. Notably, there was no correlation between Reptin and MMR mutation in CHOL and DLBC, and there was no correlation between Reptin and DNMT1, DNMT2, DNMT3A, and DNMT3B methyltransferases in UCS. In CHOL, Reptin was only significantly negatively correlated with m1A writer TRMT10C and m5C writer NSUN3.

In summary, although the role of Reptin was dependent on the cancer type, it is closely related to epigenetic regulation and mismatch repair mechanism in most tumors.

In addition, we explored the association between Reptin and immune cell infiltrates. The immune system can recognize and clear tumor cells under ordinary circumstances, but tumor cells can manipulate immune cells to evade the immune system’s surveillance (Muenst et al., 2016). In the tumor microenvironment, cancer-associated fibroblasts can modulate the function of tumor-infiltrating immune cells and affect cancer development (Chen and Song, 2019). Tumor-infiltrating lymphocytes have been identified as an independent predictor of survival and cancer sentinel node status (Santos et al., 2019). We found a negative correlation between Reptin and the infiltration of cancer-associated fibroblasts in BRCA, PRAD, TGCT, and THYM. There were significant negative correlations between Reptin and regulatory T cells in TGCT and THCA. We also found a positive association between Reptin and the infiltration of CD4^+^ Th1 cells in most kinds of tumors. These findings were confirmed by previous research, which reported that Reptin is required for T-cell development (Arnold et al., 2012), and Reptin, associated with GATA-binding protein 3, inhibits the expression of the cyclin-dependent kinase inhibitor 2c to help the cell proliferation of Th2 (Hosokawa et al., 2013). We also collected sixty immune checkpoint-associated genes in these immune cells. The results indicated significant negative correlations between Reptin and numerous immune regulators in most tumors. However, Reptin levels did not correlate with the sixty immune checkpoint genes in CHOL. Based on these findings, we infer that BRCA, PRAD, TGCT, THYM, and THCA may be suitable for anti-Reptin immunotherapy, while CHOL was not.

We also integrated the data on Reptin binding partners and Reptin interacting genes for enrichment analyses. We found that two critical genes, RUVBL1, and PIH1D1, both bind and interact with Reptin, indicating their uniqueness and importance for Reptin among numerous genes in tumors. This result was consistent with previous reports (Mao and Houry, 2017). We identified the spliceosome, Hippo signaling pathway, DNA replication pathway, and acetyltransferase activity-associated functions involved in the impact of Reptin on the tumor.

In short, we confirmed that the upregulation of Reptin is related to a worse prognosis in most tumors. It is associated with genome heterogeneity, MMRs, methyltransferase, RNA modification genes, immune cell infiltration, and immune checkpoint genes in certain tumors. Although these findings highlight Reptin as a vital cancer regulator among numerous genes and prove its potential prognosticator value and therapeutic target role for specific tumor types, further experimental validation, and mechanistic studies are needed.

## Data Availability

The dataset supporting the conclusions of this article is available from the public databases. These data can be found in The Cancer Genome Atlas (TCGA, https://tcga-data.nci.nih.gov/tcga/) and The Genotype-Tissue Expression (GTEx, https://commonfund.nih.gov/GTEx).
